# Editorial Perspective: A systems approach to addressing young people's mental health

**DOI:** 10.1111/jcpp.14077

**Published:** 2024-11-25

**Authors:** Tim Hobbs, Vashti Berry, Peter Fonagy

**Affiliations:** ^1^ Dartington Service Design Lab Buckfastleigh UK; ^2^ NIHR Applied Research Collaboration South West Peninsula (PenARC) University of Exeter Exeter UK; ^3^ Division of Psychology and Language Sciences University College London London UK

## Abstract

This editorial explores how adopting a social determinants and systemic perspective can enhance preventative measures to boost the mental health of young people. It argues that to effectively elevate the mental health of young people, it is essential to tackle both the overarching influences and their specific local impacts. We maintain that a strategy combining systems thinking with evidence tailored to the local environment and participatory design is essential.

## A lack of progress in improving young people's mental health and well‐being

In general, the mental health of children and young people is deteriorating: the prevalence of many mental health disorders is increasing, well‐being is decreasing, and inequalities in mental health are widening for some groups (Castelpietra et al., 2022; Newlove‐Delgado et al., 2022).

Over the past two decades, there has been significant investment in research, design, and delivery of mental health therapies, as well as in the provision of psychological and psychiatric services (Cohen, 2017; Insel, 2022). This growth has been spurred in part by rapid and significant developments in the life sciences, including neuroimaging research now being enhanced by artificial intelligence techniques, computational psychiatry, and studies in genomics (polygenicity) and gene–environment interactions (Gandal & Geschwind, 2021), including DNA methylation (Zhang et al., 2023), which contribute to the field of precision psychiatry (Kambeitz‐Ilankovic, Koutsouleris, & Upthegrove, 2022). These advances have enriched our understanding of risk factors and hold the potential to refine our strategies for early identification and the design and implementation of targeted, indicated, and universal interventions (psychotherapeutic, pharmacological, and digital health technologies). Recent innovations have introduced promising approaches that are poised to transform psychiatric practices and research. These include clinical neuroscience and personalised drug therapies; cutting‐edge statistical methods for psychiatric classification, assessment, and research; a shift towards community mental health care and away from institutionalisation; broader access to evidence‐based psychotherapy; the use of digital tools for symptom tracking and therapy; and global mental health initiatives and task‐sharing strategies in care management (Stein et al., 2022). Upon detailed examination, while these innovative approaches provide valuable insights and generate optimism for further developments, we concur with Stein et al. (2022) that expectations for dramatic changes in the field are not convincingly substantiated. There has been noticeable progress in the diagnosis and treatment of mental disorders in recent years, reflecting increased investment, but these changes have yet to significantly reduce the overall burden of mental disorders.

Part of the reason for this may be that mental health investment is heavily skewed towards individual treatment services, narrowly defined health outcomes, and mono‐causal explanations. Despite increased funding, the effectiveness of interventions has shown little improvement, if any (NICE, 2022), and despite more treatment options and availability, the prevalence of mental ill health has continued to rise (Ormel, Hollon, Kessler, Cuijpers, & Monroe, 2022). Current strategies may be inadequate because they largely focus on diagnosing specific disorders, emphasising ‘risks’ and ‘shortcomings’ linked to each condition. Yet, the reality often involves complexities and co‐occurring conditions (Colizzi, Lasalvia, & Ruggeri, 2020), and there is a deficiency in evidence‐based guidance for effectively pinpointing the causes of psychological disorders at an early stage.

In other words, many of our treatment services and pathways are designed to address specific symptoms or conditions, such as anxiety, while a myriad of intersecting needs, experiences, and underlying or comorbid conditions might go unaddressed by overextended mental health professionals. This oversight can lead to a cycle where economic instability and housing issues increase stress, which may then trigger poor immune responses and sickness, leading to time off work and perpetuating poverty. Approaches to tackle these issues, and understanding what interventions are effective for whom, and why they work, are currently lacking.

In brief, regrettably, the focus on diagnosis and treatment has not been matched by adequate attention and investment in addressing the structural and social determinants of mental health and well‐being, including health promotion and preventative policy and practice opportunities (Fried & Robinaugh, 2020; Marmot, Allen, Boyce, Goldblatt, & Morrison, 2020).

## Social determinants of young people's mental health: A systems issue

It is now widely acknowledged that a variety of demographic, social, cultural, economic, neighbourhood, and environmental influences interact to affect young people's mental health (Compton & Shim, 2015). These factors also affect the accessibility, effectiveness, and impact of services and support systems (Lund et al., 2018). These social determinants reciprocally drive and are fuelled by social inequalities, poverty, and deeply entrenched systemic discrimination (Alegría, NeMoyer, Falgàs Bagué, Wang, & Alvarez, 2018).

Given the multitude of interacting influences, we and others argue that young people's mental health and well‐being should be considered a ‘systems issue’ (Compton & Shim, 2015; World Health Organization (WHO) 2022). This perspective builds on the long‐established social and ecological models of health (Bronfenbrenner, 1994; O'Connor, 1977) and further considers mental health and well‐being as a dynamic state that varies over time and is influenced by the interactions of these broader social determinants (Cohen, 2017).

## Implications for prevention

In wider fields of public health, systemic change efforts often focus on macro‐system policy levers such as poverty, economic inequality, employment, housing, and transport (Marmot et al., 2020). There is considerable potential for impact by initiating changes at this level (Bolton, 2022; Meadows, 1999) and redirecting resources and system structures towards prevention (Colizzi et al., 2020; Uhlhaas et al., 2023). There is evidence that action across the policy areas of health systems, youth support systems, workplaces, and welfare systems can enhance the health, educational and labour market opportunities and outcomes of individuals experiencing mental health issues (Britteon et al., 2024; Knapp, McDaid, & Parsonage, 2011; OECD, 2021). However, sustained policy change is challenging due to macro‐economic contexts, shifting political priorities, short‐term budget cycles and pressure for ‘quick wins’.

Furthermore, the ways in which social determinants influence young people's mental health will vary considerably depending on local context, individual circumstances, and their interaction within local settings (Alegría et al., 2018). For example, in rural or coastal regions, factors such as social isolation, poor transport infrastructure, and limited opportunities for meaningful employment may significantly impact mental health, whereas in diverse and densely populated urban areas, issues related to safety, racism, and discrimination may be more significant influences (Santana de Lima et al., 2023).

As such, we contend that alongside considering macro‐influences, it is crucial to adopt a more nuanced local perspective, examining how the social determinants of mental health manifest at the micro/local level—a principal focus of many local health systems, including the newly established Integrated Care Systems (ICSs) in England.

High‐quality local epidemiological data on young people's mental health and well‐being is essential for understanding the role and impact of social determinants at the local level. Although not widely available, when such data are collected and utilised effectively, they can elevate the profile of young people's mental health on the local policy agenda, shape local policies, and encourage change (Marquez, Humphrey, Black, & Wozmirska, 2024).

Furthermore, like other commentators (Baxter, Barnes, Lee, Mead, & Clowes, 2023; Leung, 2004), we argue that the voices of young people and community representatives are crucial not only in interpreting such quantitative data but also in qualitatively articulating how the social determinants of mental health are understood and expressed systemically at the local level, how inequalities are experienced by different groups, and how these insights might be leveraged. These types of data and local insights can then be used to inform the systemic co‐design of health‐promoting and preventative policies and practices directly addressing the social determinants of young people's mental health at the local level.

Over the past decade, there has been an increasing interest in and application of youth‐centred co‐design approaches, which are informed to varying extents by existing research evidence (Blanchard & Fava, 2017; Lyon, Brewer, & Areán, 2020; Lyon & Koerner, 2016). However, the majority of these co‐design efforts tend to concentrate on early intervention and treatment service design rather than on preventative approaches that target the social determinants of young people's mental health. To shift the focus of co‐design efforts towards the social determinants of mental health, it may be beneficial to incorporate a variety of systems thinking methods. Examples include community‐based participatory group model building, where young people, community partners, practitioners, and commissioners collaboratively develop system maps of key systemic influences, thereby identifying intervention points and areas for leverage (Hovmand, 2014; Savona et al., 2023).

For example, as part of the Kailo initiative (Hobbs, Santana De Lima, Bevington, et al., 2023), local community partners in a rural/coastal area of England explored existing data and undertook an extensive process of engaging young people and community partners to explore the social determinants of young people's mental health and well‐being in that context. This uncovered a range of contextually specific influences and inequalities, including, for example, geographical and social isolation (compounded by poor transport and social infrastructure), a lack of awareness of diverse vocational and employment opportunities (beyond agriculture and tourism), and limited knowledge and awareness of youth mental health and informal sources of support from adults in the community, in turn eroding a sense of young people's identify and belonging (Santana de Lima et al., 2023). This locally nuanced and systemic exploration of inequalities and the social determinants of mental health has informed local policy and strategy formulation and driven the coalescence of youth‐centred co‐design teams to start designing local responses to the challenges that surfaced. These have included, for example, the co‐design of youth‐centred and community‐based youth employment hubs and input to the design of local youth mental health and advisory hubs (with research also feeding into central government economic stimulation and transport infrastructure building as part of ‘levelling up’ capital investments).

## Conclusion

To further advance prevention science related to young people's mental health, we advocate for a deeper exploration of how macro‐determinants of mental health manifest at the local level. We see considerable potential for such systemic, youth‐ and community‐centred approaches to exploring the manifestation of the social determinants at the local level, forming a strong foundation for co‐designing contextually relevant services, policies, and community preventative responses that address the social determinants most pertinent to young people's mental health and well‐being in local contexts.

This requires long‐term, sustained and evolving partnerships—ideally underpinned by place‐based or pooled budgets—to build strategically aligned portfolio‐based approaches (i.e. moving beyond single or isolated interventions to the design of place‐based, coordinated strategies comprising a suite of mutually reinforcing policy and practice interventions).

We are beginning to implement such ideas and approaches as part of the UK Prevention Research Partnership‐funded initiative, Kailo, and will share insights from an accompanying developmental and impact evaluation as they become available (Hobbs et al., 2023). We encourage others to explore and test approaches that address the social determinants of young people's mental health, to start demonstrating the effectiveness of preventative strategies in response to increasing needs and the unsustainable demand for costly treatment interventions.

## Data Availability

Data sharing is not applicable—no new data generated, or the article describes entirely theoretical research.
